# Supplementary material for: A new experimental platform facilitates assessment of the transcriptional and chromatin landscapes of aging yeast
Source: eLife. 2018 Oct 19;7:e39911. doi: 10.7554/eLife.39911 (PMC6261268; doi:10.7554/eLife.39911)
Supplement: Figure 1—source data 3. [file elife-39911-fig1-data3.pdf]

Transcript classification	Abbreviation	Reference/Definition
meiotic unannotated transcripts	MUTs	Lardenois A, et al. (2011)
Xrn1-sensitive unstable transcripts	XUTs	E. L. van Dijk, et al. (2011)
cryptic unstable transcripts	CUTs	Davis CA, Ares M Jr. (2006),Xu Z, et al. (2009)
stable unannotated transcript	SUTs	Xu Z, et al. (2009)
Antisense	AS	Yassour 2010
Extended 5’ 3’ UTRs	UTRs	Johnson 2011,Nagalakshmi 2008, Yassour 2009, Arriberre (2013)
TY repeat elements	TY	Transposons & long terminal repeats as defined by SGD (gbrowse track)
Subtelomeric	TELO	< 40 Kb from chromosome end
