# Supplementary material for: A new experimental platform facilitates assessment of the transcriptional and chromatin landscapes of aging yeast
Source: eLife. 2018 Oct 19;7:e39911. doi: 10.7554/eLife.39911 (PMC6261268; doi:10.7554/eLife.39911)

**Supplementary File 1**

**MAD System Setup**

Ver 1.0 / 2018

**1. Introduction to MAD System**

**2. Building up MAD System**

**3. Preparation of MAD Components**

**4. Media Prep and Assembly of MAD Components**

**5. Running MAD System and Daily Monitoring**

**6. Disassembly of MAD System and Cleanup**

**Bolded items** are found in the excel sheet listing materials.

**1. Introduction to MAD System**

This manual is to introduce MAD (**M**iniature-chemostat **A**ging **D**evice) system to enrich aging yeast mother cells in, and to give detailed instruction to build, assemble, operate, and cleanup the MAD system and components. MAD system is based on ministat system developed in Dunham lab (hereafter ‘ministat’), and if you are new to chemostat or ministat, it is desirable to read comprehensive manuals about chemostat or ministat operation by the Dunham lab listed below.

Chemostat manual <http://dunham.gs.washington.edu/Dunhamchemostatv2.pdf>

Ministat manual <http://dunham.gs.washington.edu/DunhamLabMinistats.pdf>

Ministat paper <http://www.jove.com/video/50262/>

The MAD system described here contains up to 8 **vessels**. Depending on the design of each experiment, this system can be operated with only a few **vessels** or can be expanded to higher number of **vessels**. Note that the MAD system is designed to operate in a *temperature controlled warm room*. If a warm room is not available, a **dry heat block** can be used.

There are a couple of modifications between a normal ministat and a MAD. First, in the MAD setup, each **vessel** gets its own **air pump**. The **air pump** used here has only one outlet and the air pressure can be adjusted with a controller to ensure optimum air flow that is strong enough to generate turbulence in the **vessel** (aeration and stirring), but weak enough not to disturb labeled aging yeast cells attracted to magnetic fields. For finer control of air pressure, an **air pressure gauge** can be connected to each air pump (Section 2-2 in this manual). Second, tubes for media and air are merged with a **Y-connector** before being connected to the **vessel** (Section 2-5-a and 3-1-j in this manual). In this way, an air humidifier is not required, and a more continuous supply of media is guaranteed. Third, a “loading port” is introduced to prevent contamination possibly generated during inoculation in the ministat system. Lastly, the MAD system uses several custom components. These include ring magnets, an air-bubble trapper, Teflon cap, and a silicon disc gasket, which are fully described in the following sections.

**2. Building up MAD System**

**1) Metro Rack**

**Metro rack** (48X24X63”) with three shelves houses the MAD system.


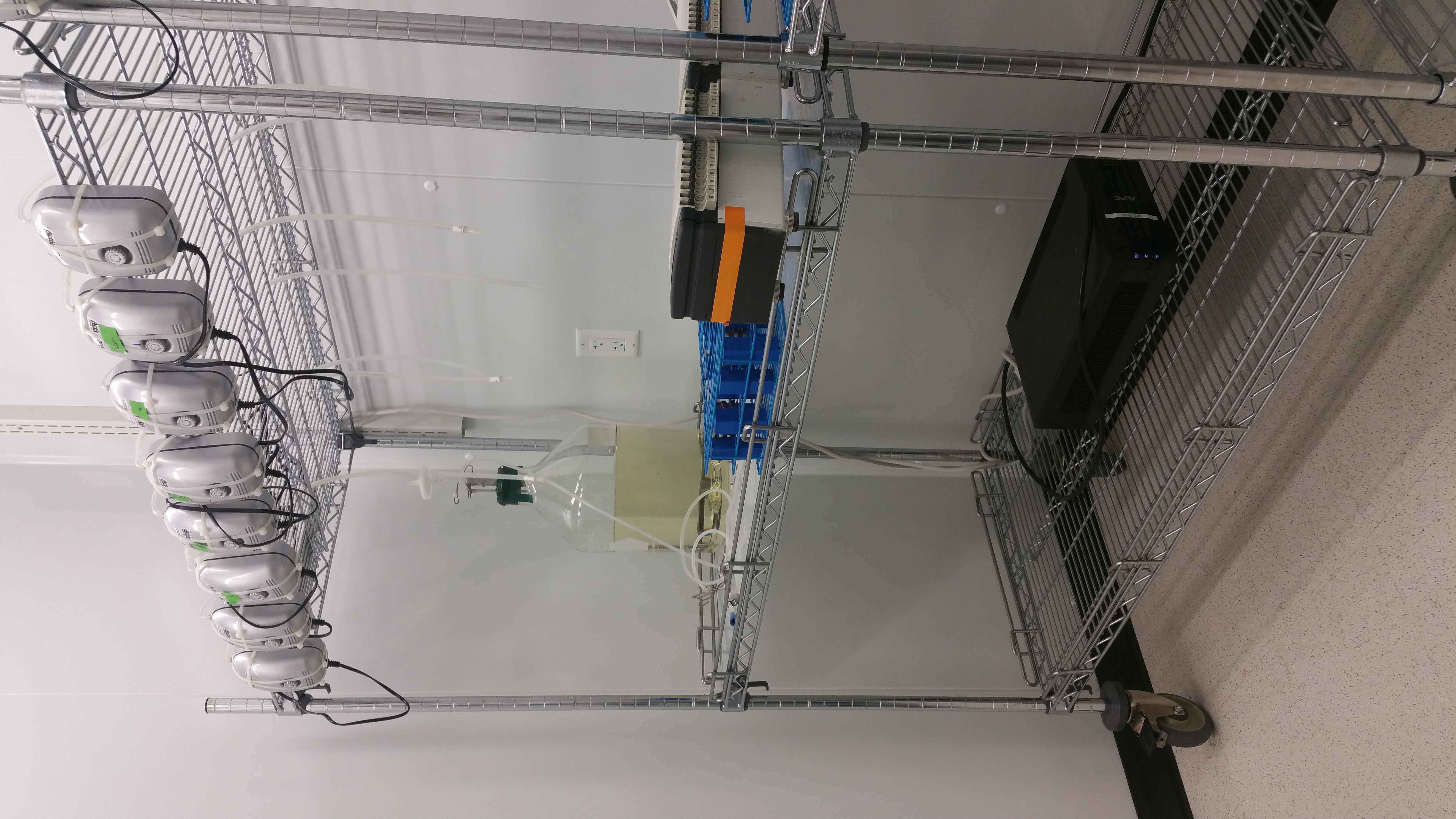
 **
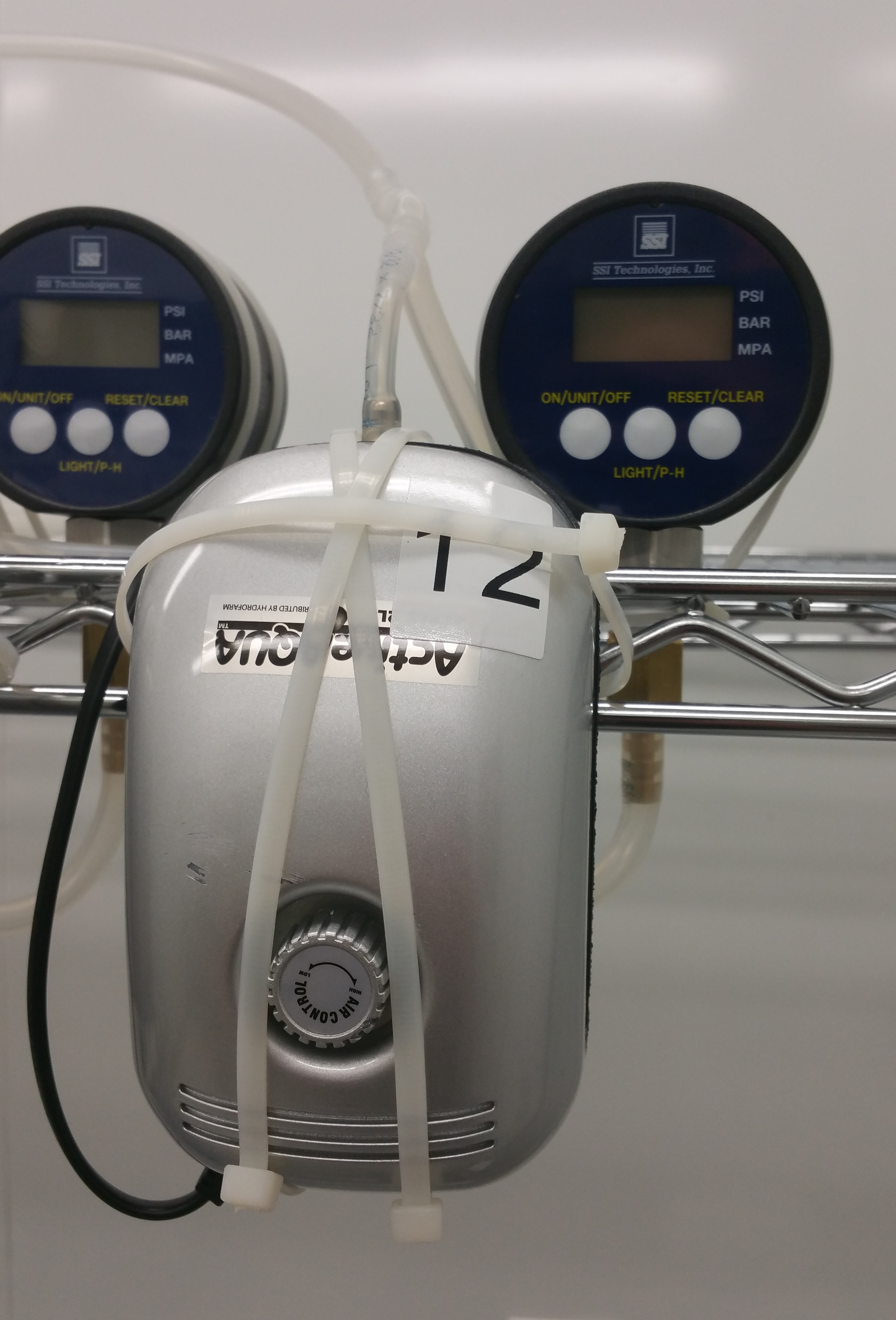
**

Air pump with air pressure gauge

Metro rack with 3 sheves

**2) Air pumps**

**Air pumps** are mounted on the front side of top shelf with **long cable ties**. A piece of **small tubing** (1 inch long) is connected to the outlet of each **air pump** and fastened with a **short cable tie** (see above). A **small reducing connector** is used to connect to **small tubing** with **medium tubing** (1.5” long).

**3) Ring magnets in a rack**

**Neodymium ring magnets** are extremely strong and may cause injuries to your fingers when you stack them (be sure to handle carefully). These **magnets** may also lead to malfunction of electronic devices. Three stacked ring **magnets** will generate strong enough magnetic fields for most applications. Once these **magnets** are stacked without spacers, it is extremely difficult to separate them again, and since the spacers that come with the magnets from manufacturer do not fit to the **vessels**, we made custom **magnet spacers** with the same dimensions as the **magnets**. Carefully separate three magnets from a package, move them apart from one another, and restack the **magnets** with two **magnet spacers**. Insert the stacked **magnets** in a metal **tube rack** and insert **magnet holders** as shown below. **Magnet holders** are to prevent stacked **magnets** from bumping to the next ones.

magnet spacer

magnet holder

3 stacked ring magnets in a tube rack with magnets spaces and holder


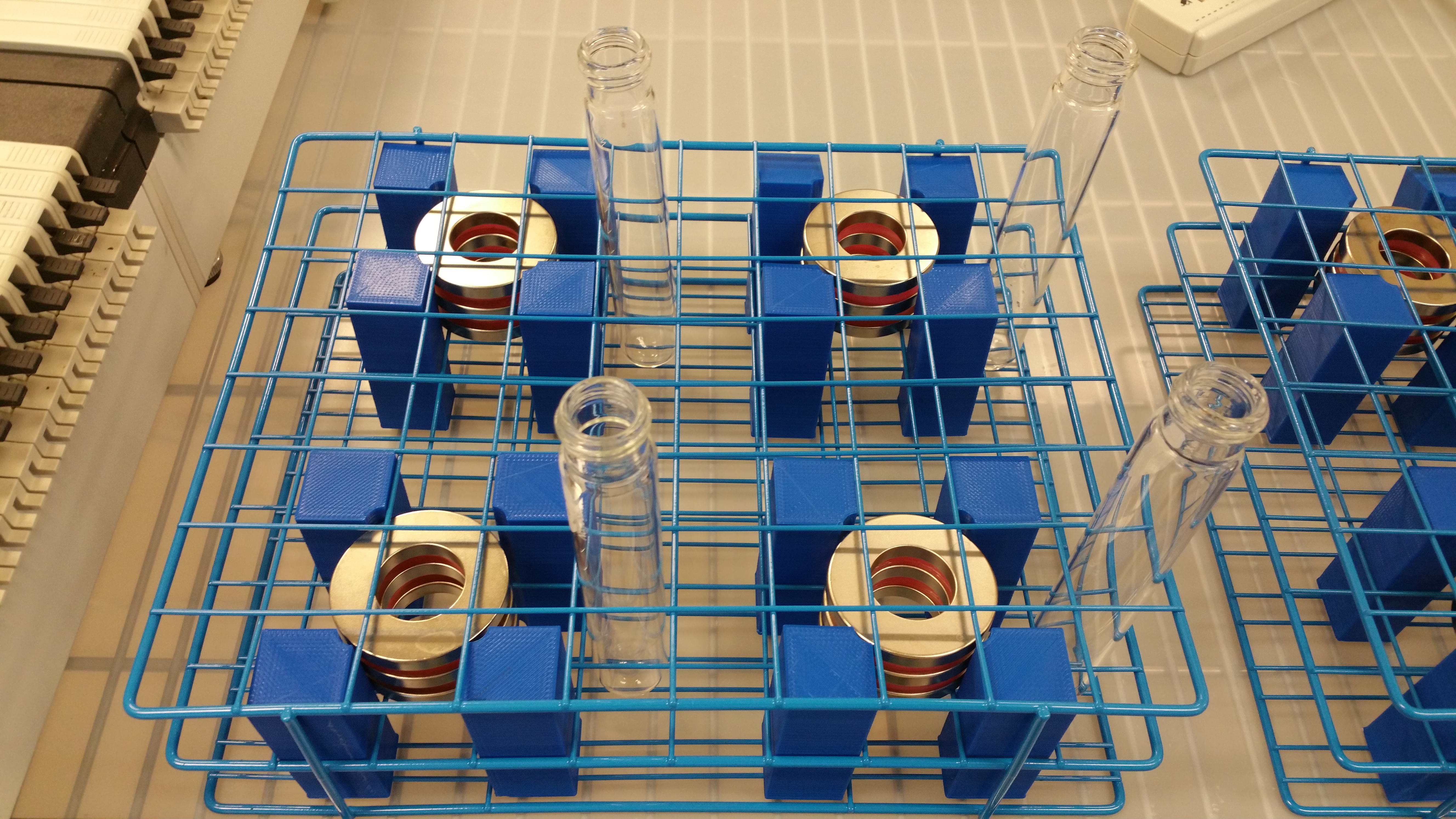


**4) Air Bubble Trapper (“Bubble Cage”) and Teflon Cap**

It is critical to guide air bubbles generated from the **air pump** through a **long needle** in the center of the **vessel** to not disturb labeled aging yeast cells. To this end, an **air bubble trapper** (or “bubble cage”, rod + tube) has been designed as described below. The **air bubble trapper tube** is to trap the air bubbles generated from the air pump, such that only small bubbles can escape. The **air bubble trapper rod** is to position a long needle in the center of the vessel. The **Teflon cap** with **silicon disc gasket** are used to help guide the needles and to make the vessel airtight. These **air bubble trappers**, **Teflon caps**, and **silicon disc gaskets** can be purchased from Zera Development (Santa Clara, CA) (see Table S4 for catalog numbers).

1. Specifications for **air bubble trapper rod**

- Material: 316 Stainless Steel (McMaster-Carr 9298K12)
- Length: 1¼ inches
- Outer diameter on top : ½ inches
- Outer diameter on bottom with outer thread: ^13^/_32_ inches
- Length of outer thread on bottom: ^7^/_16_ inches
- Length of non-threaded top: ¾ inches
- Length between outer thread on bottom and non-threaded top: ^1^/_16_ inches
- Diameter of center hole : ^1^/_16_ inches

1. Specifications for **air bubble trapper tube**

- Material: 316 Stainless Steel (McMaster-Carr 89495K425)
- Length: 5 inches
- Outer diameter: ½ inches
- Wall thickness: 0.065 inches
- Length of bottom area: ½ inches
- Length of area with holes: 2¾ inches
- Length of top area: 1¾ inches
- Length of inner thread: ^7^/_16_ inches
- Number of holes: 16 X 16
- Diameter of holes: ^3^/_32_ inches


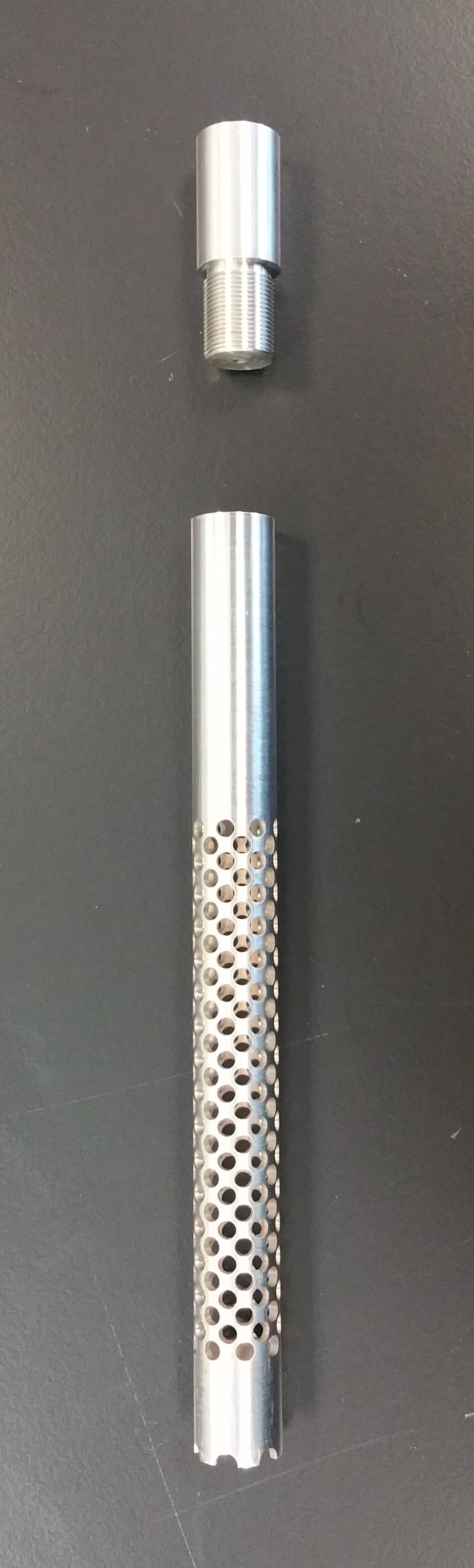

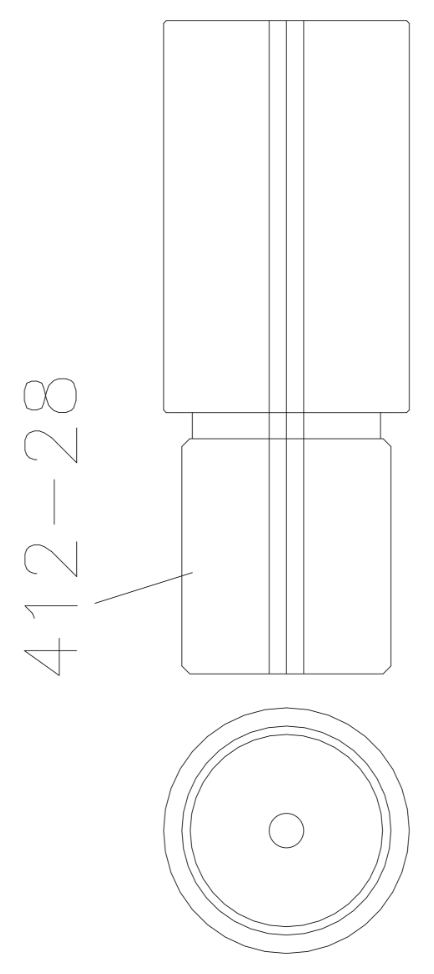

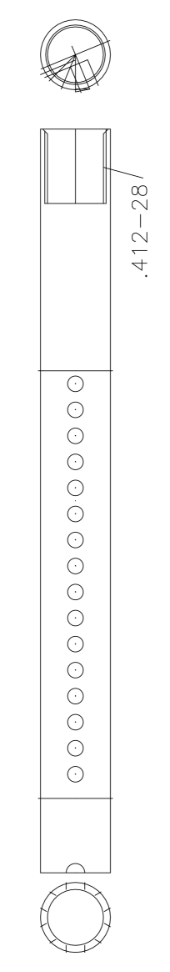


air bubble trapper rod

air bubble trapper tube

air bubble trapper rod

air bubble trapper tube

1. Specifications for **Teflon cap** and **silicon disc gasket**

- Material: McMaster-Carr 8546K17 - Material: McMaster-Carr 1460N25
- Outer diameter: 1¼ inches - Diameter: 7/8 inches
- Length: 1^5^/_16_ inches - Thickness: 3/16 inches
- Diameter of holes: ^1^/_16_ inches

**
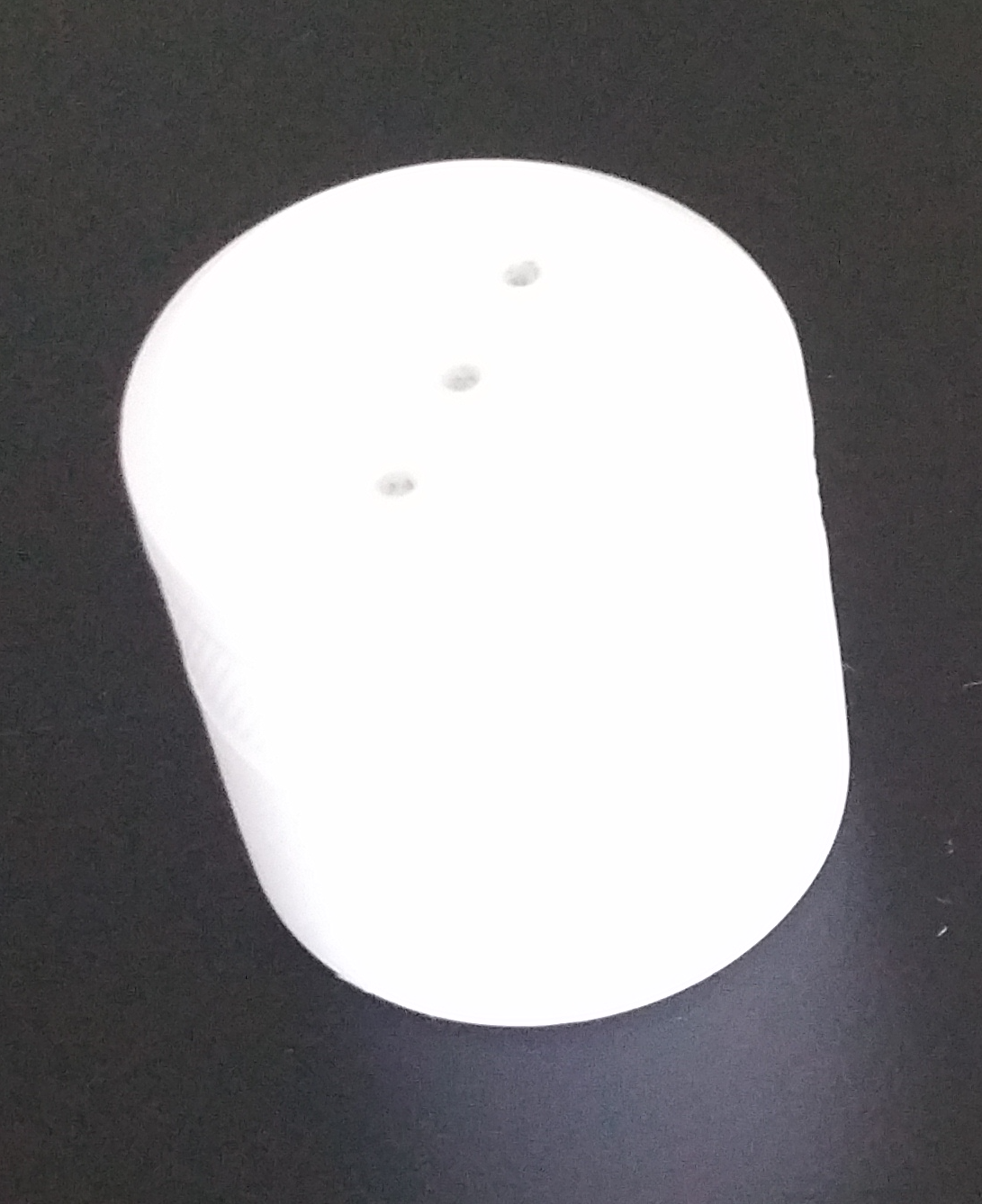
** **
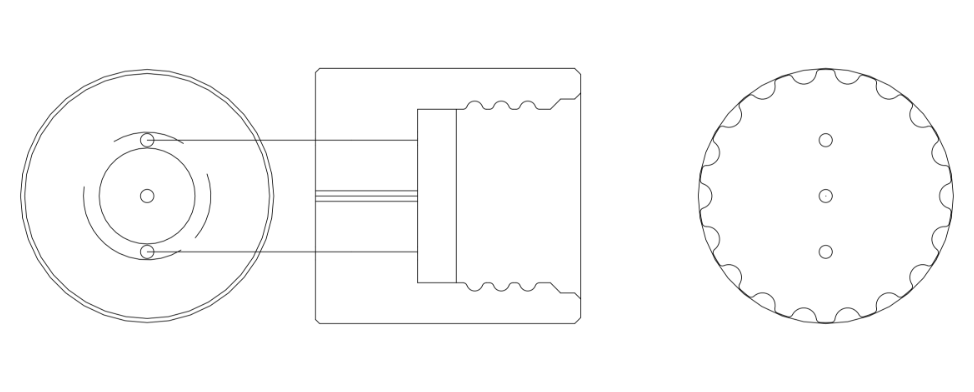
**
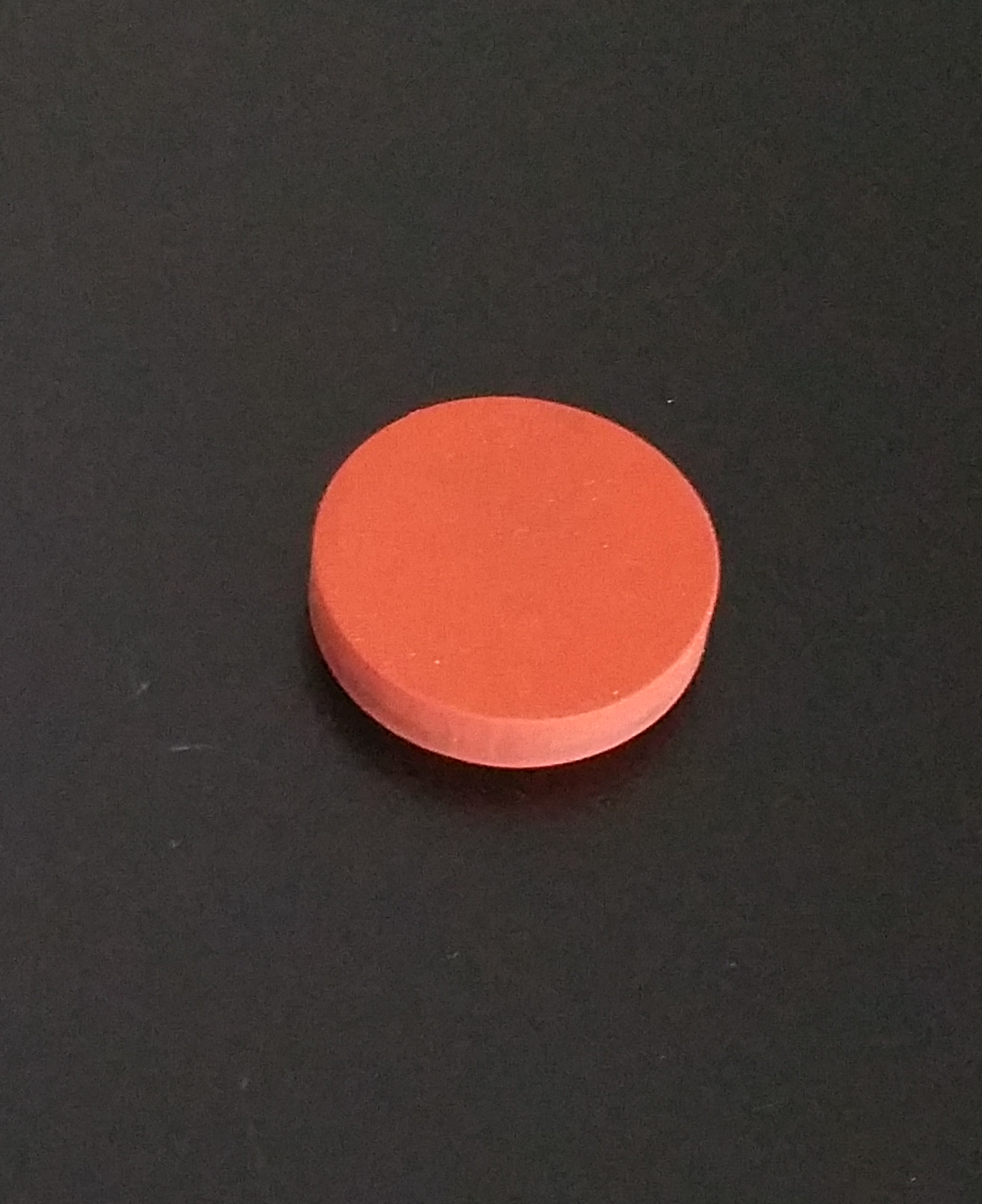


**5) Tubing information**

a. **media/air tubing**: This tubing is composed of a 16-inch long small tube (**air tubing**) and 20-inch long small tube (**media tubing**) combined to a 1-inch long small tubing via **Y-connector**. At the end of the 1-inch small tubing (and at the other side of media tubing), insert male luers. Plug a syringe filter at the end of air tubing. Air tubing should be long enough to reach to an air pump mounted on the metro rack, The **media tubing** connected to **marprene tubing** should be long enough to reach to a media carboy through the **peristaltic pump**. It is highly recommended to have same length of air, media, and connector tubing for each MAD unit.


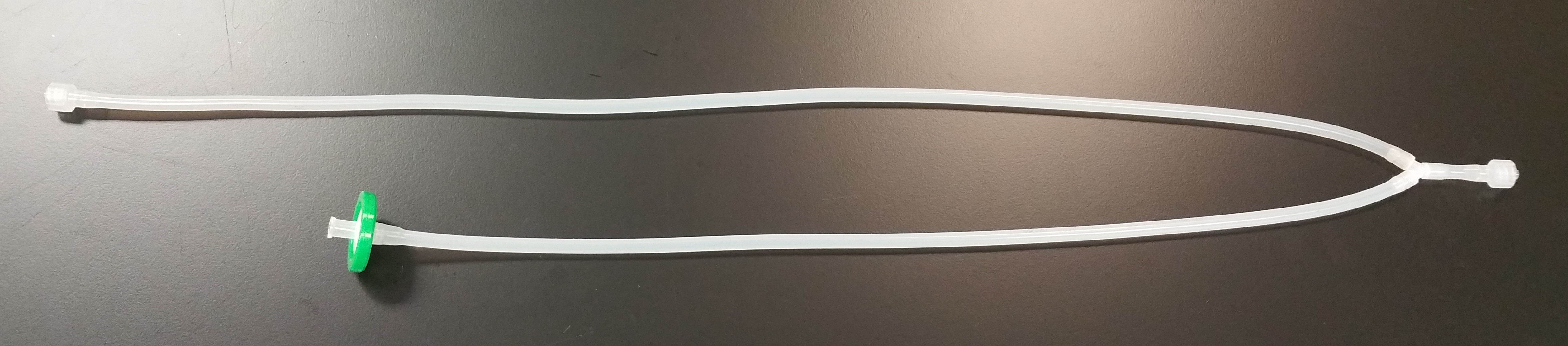


a

b. **effluent tubing**: This is a 36-inch long small tube with a male luer inserted at one end.


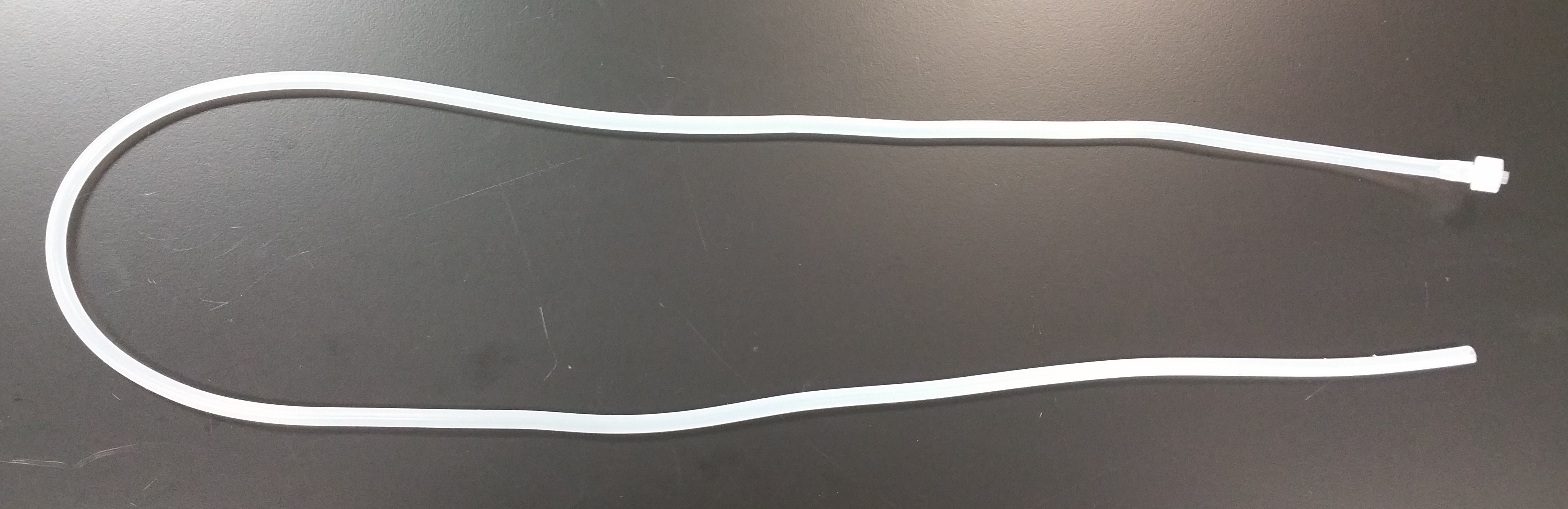


b

c. **marprene tubing** : Carefully insert a blunt-end needle at each end of marprene tubing.

**
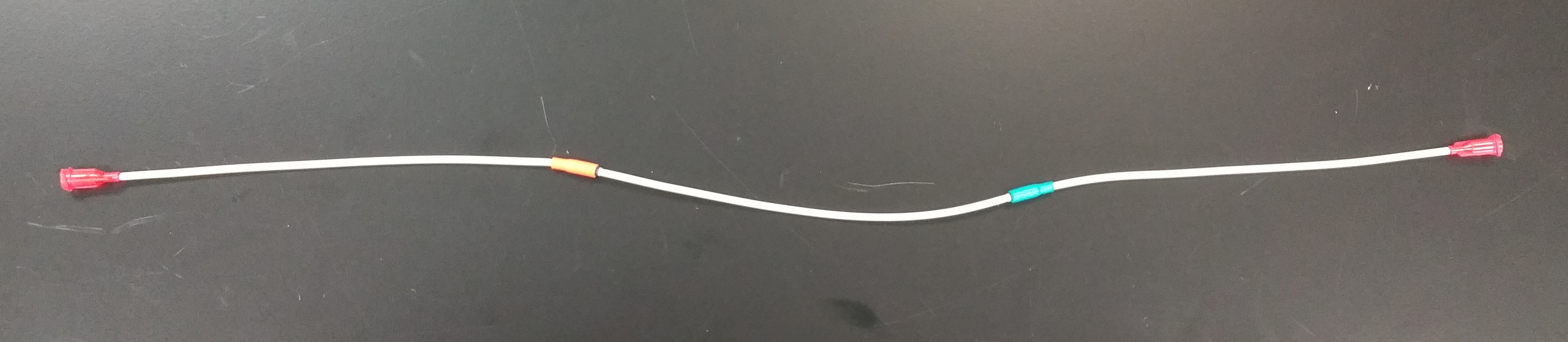
**

c

d. **connector tubing** : This is a 15-inch long small tube with a male luer and a female luer.

**
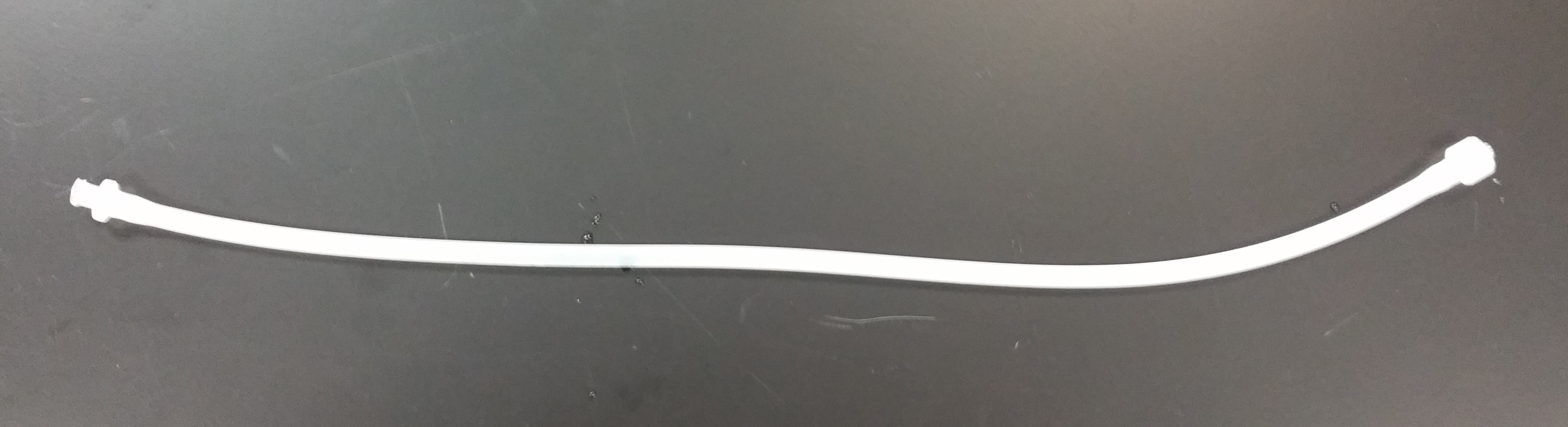
**

d

**3. Preparation of MAD Components**

**1) Preparation of Vessels and Tubing**

1. Insert a **silicon disc gasket** into a **Teflon cap** using a **flat forceps** or by hand.


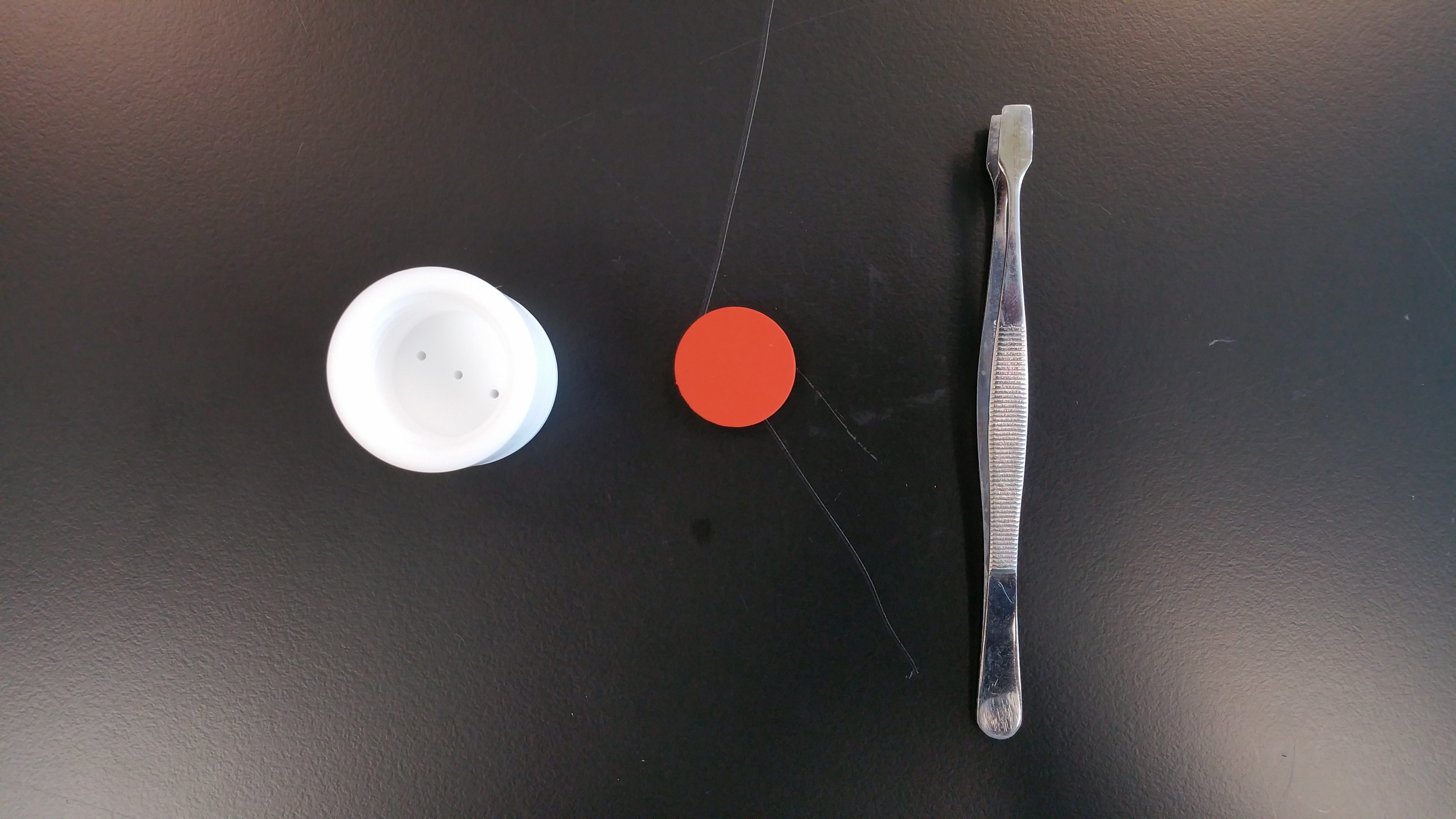

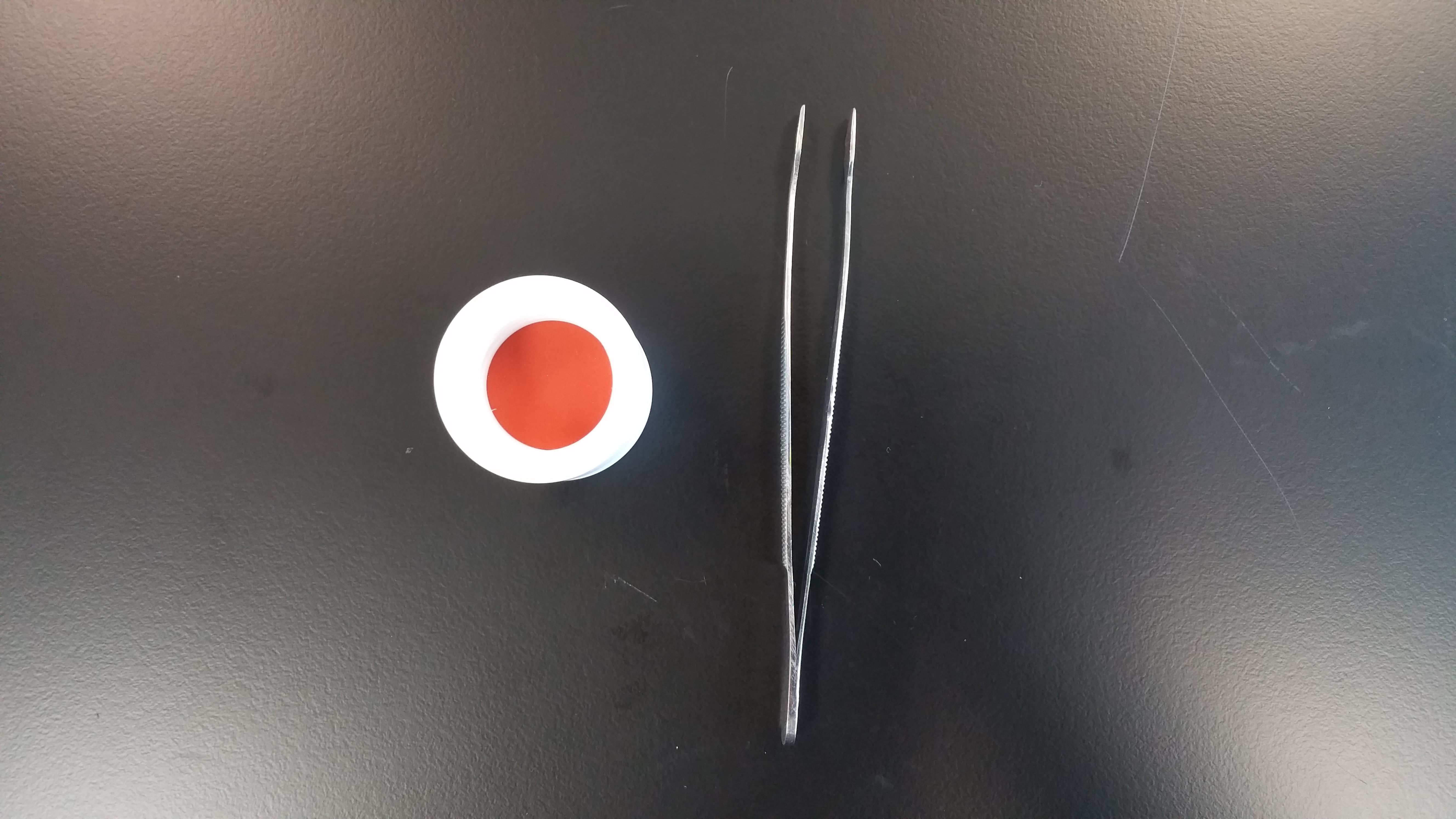


a-1

a-2

1. Screw the **Teflon cap** into glass **vessel**, and place into **tube rack**.
2. Insert a **long needle** (for media and air) into the center hole of the cap and two **short needles** (one is for effluent and one is for the adding port) ~1” through the **silicon disc gasket**.
3. Unscrew the **Teflon cap** from the glass **vessel**, and remove debris of **silicon disc gasket** with a **fine forceps**. Debris should be found at the tips of the needles, in the **vessel**, or on the **gasket** itself. A syringe can be used to test whether or not the needles are blocked with debris.


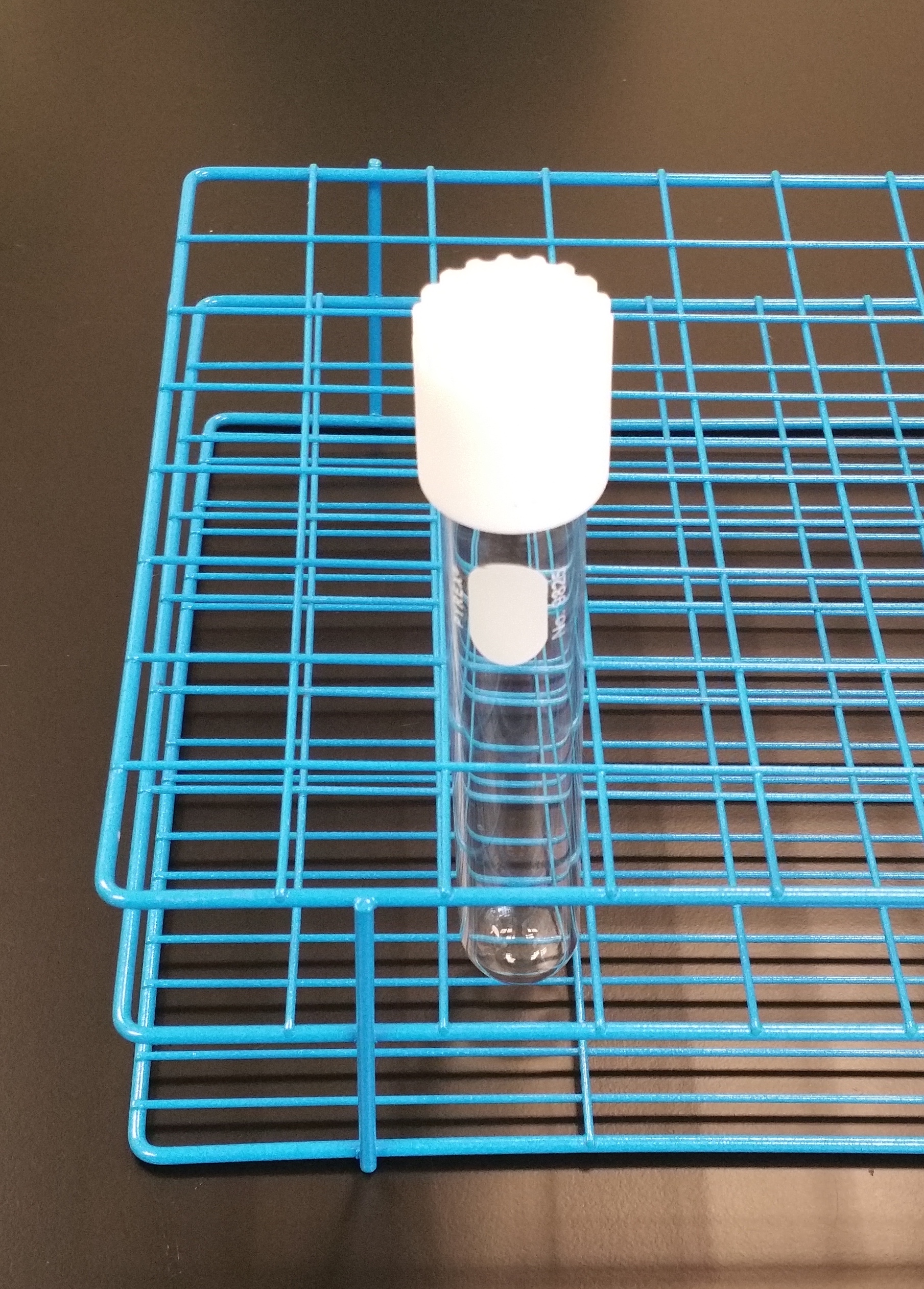

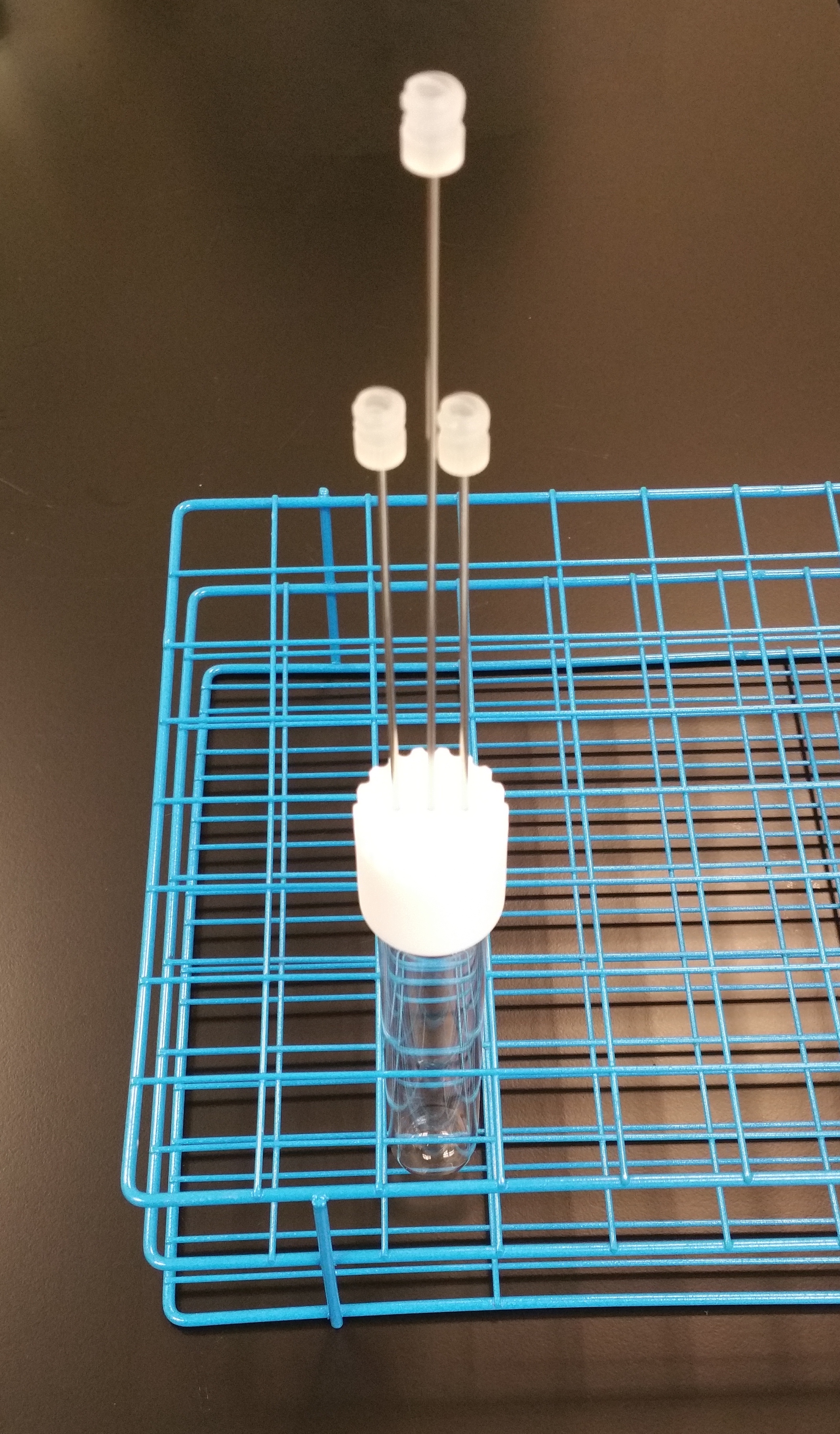

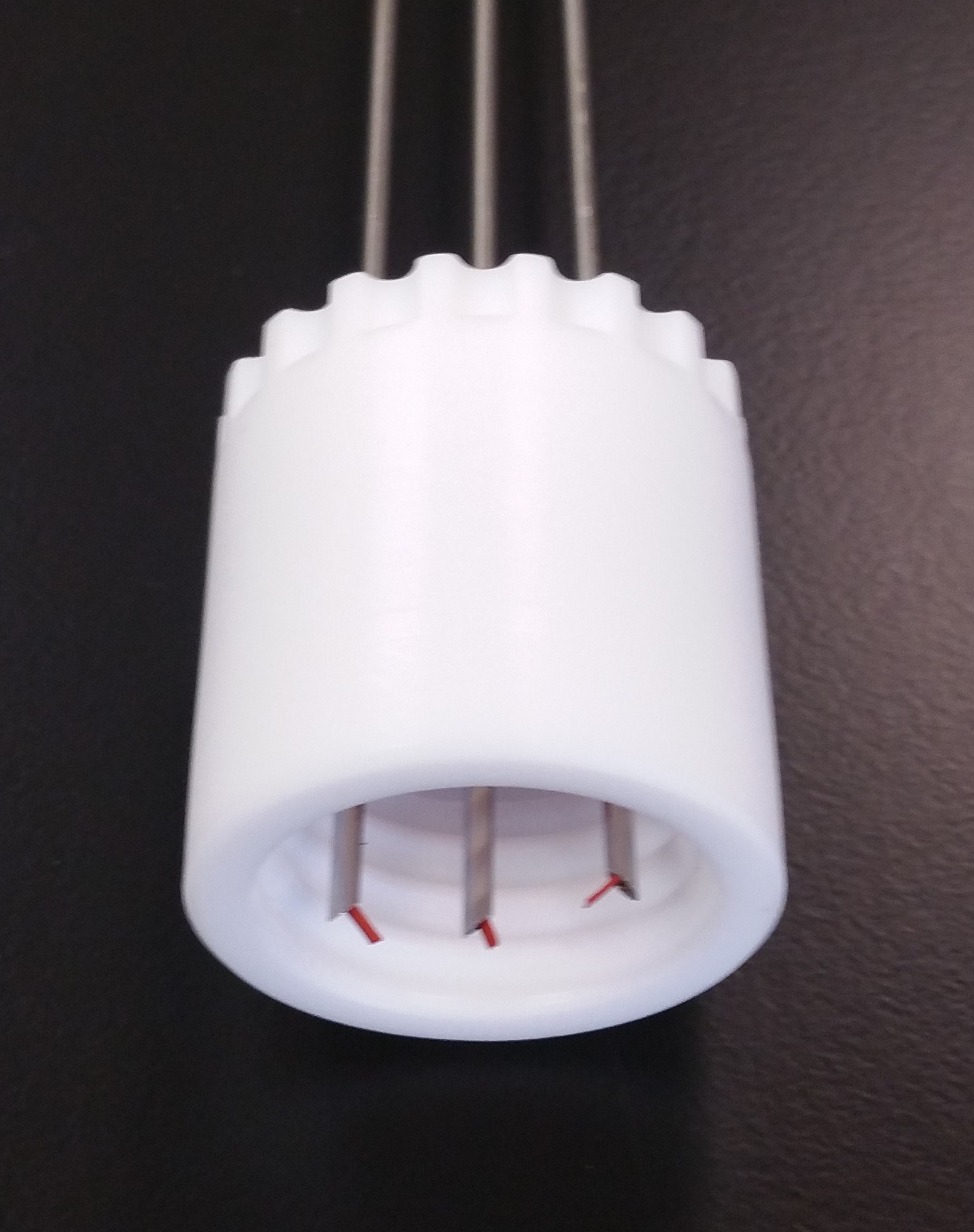

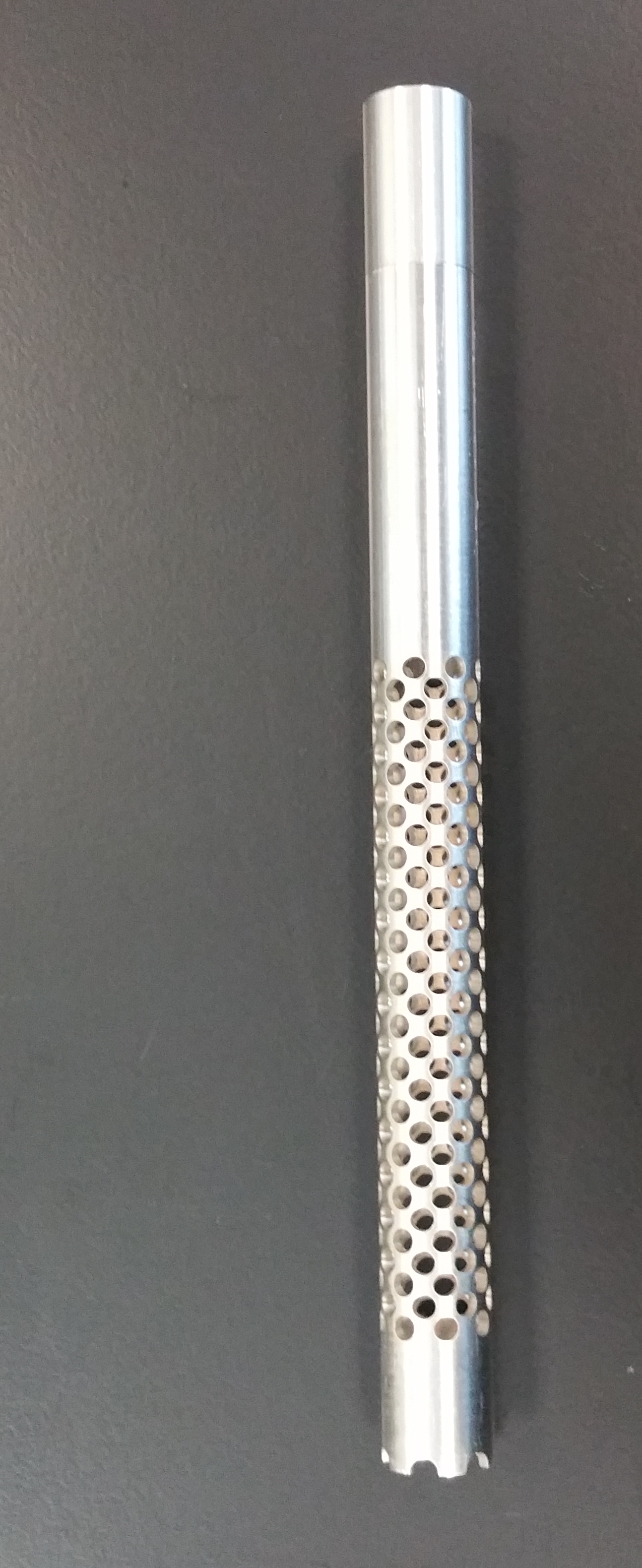


b

c

d

e

j


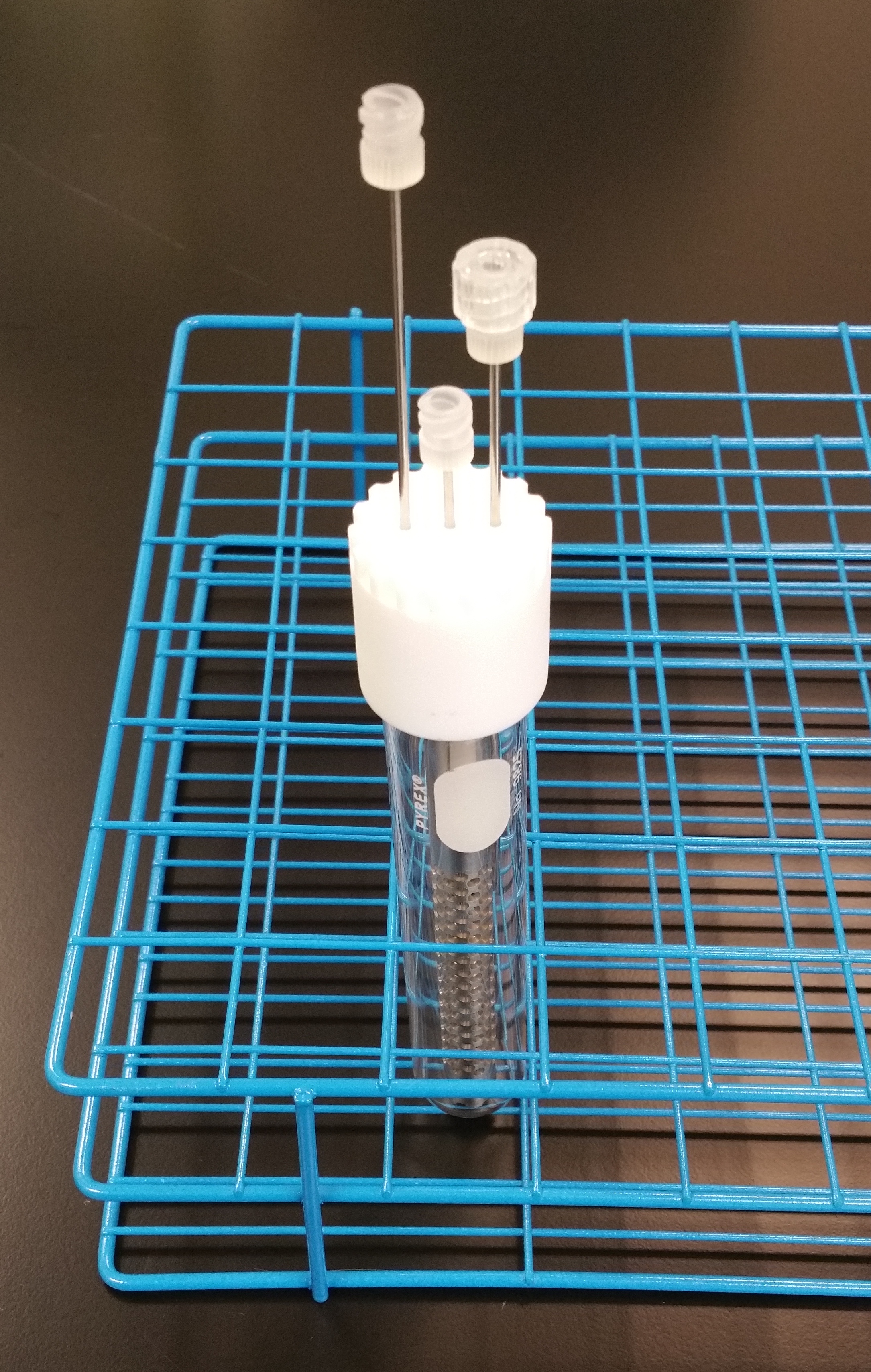

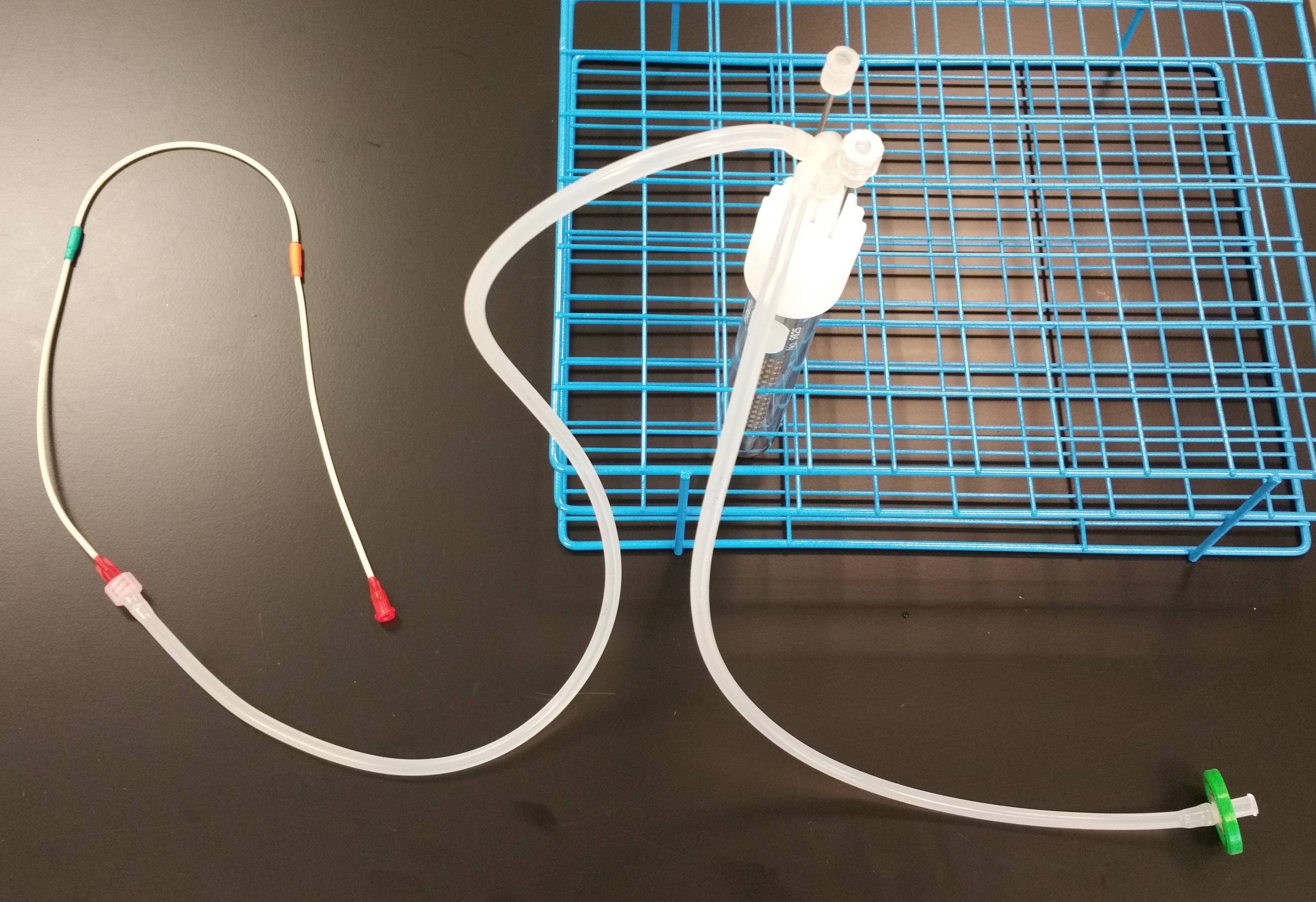


f-i

1. Screw an **air bubble trapper tube** with an **air bubble trapper cap**, and insert the **long needle** through the hole on **air bubble trapper cap**.
2. Insert the **air bubble trapper** into a glass **vessel** and attach the **Teflon cap**.
3. Push the **long needle** all the way down to the bottom of glass **vessel** and make sure the **long needle** is positioned in the center of the glass **vessel**.
4. Push down one **short needle** to the bottom of white labeling area on the **vessel**. This needle is for effluent and it will maintain the culture volume at ~40 mL.
5. Push down another **short needle** half way down. This needle is an *adding port* and attached a **male luer plug**.
6. Connect the *media/air tubing* with the center **long needle**, and **marprene tubing** to the end of *media/air tubing*.
7. Attach the *connector tubing* to the end of the **marprene tubing**.
8. Connect an *effluent tubing* to the needle for effluent.
9. Cover the ends of *connector tubing*, *effluent tubing*, and **air filter** with aluminum foil. Refer to *chemostat manual, page 19, foil origami method*.
10. Repeat the steps *a* to *m* for other vessels.
11. Put autoclave indicator tape on the **Teflon caps**, and autoclave for 12 minutes on dry cycle. Note that the vessels with tubings should be taken out immediately after autoclave. Incubation of these plastic hub of needles, luers, and plugs in autoclave at high temperature will change the shape of these parts, which may result in leaks.

k

m

l


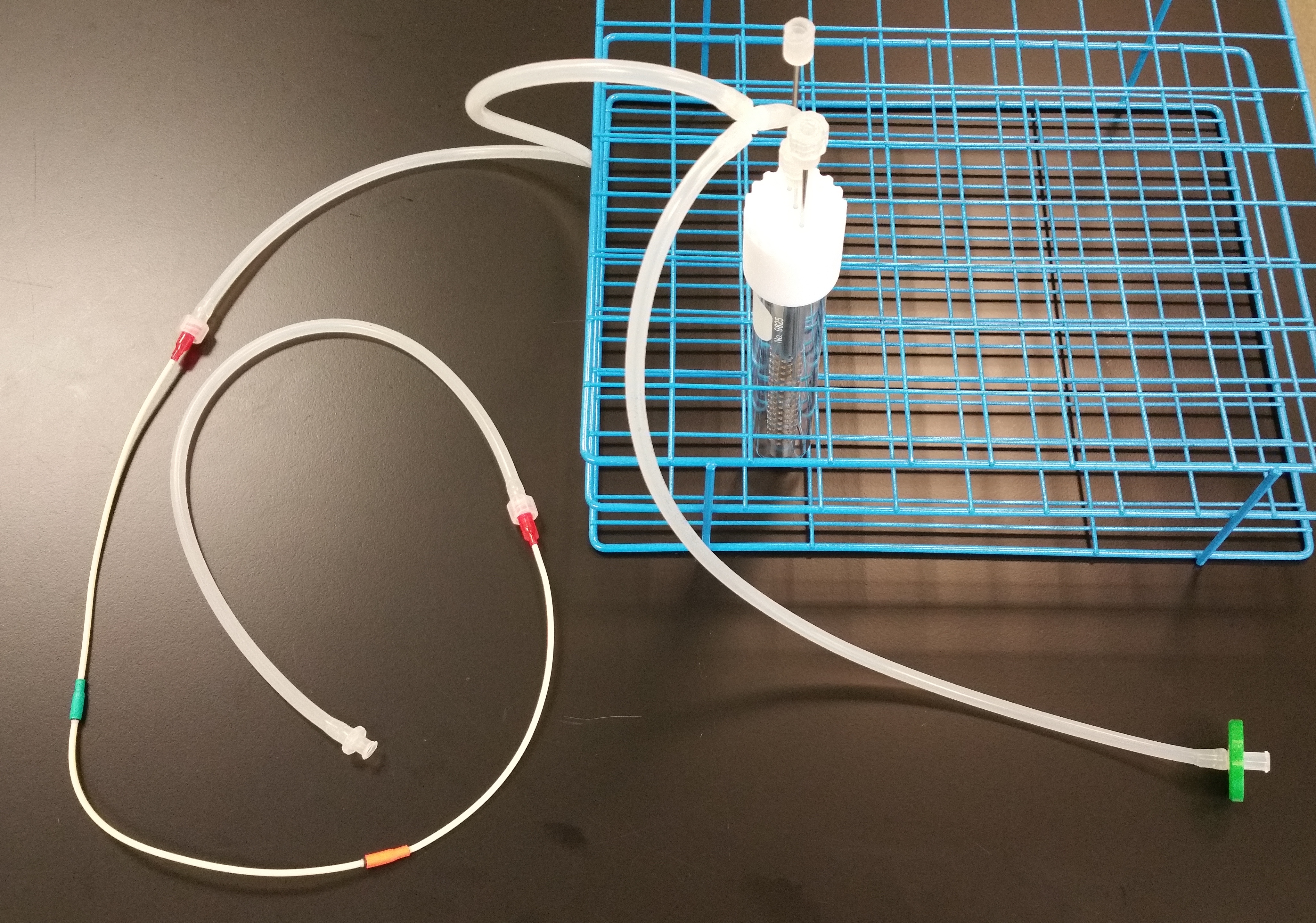

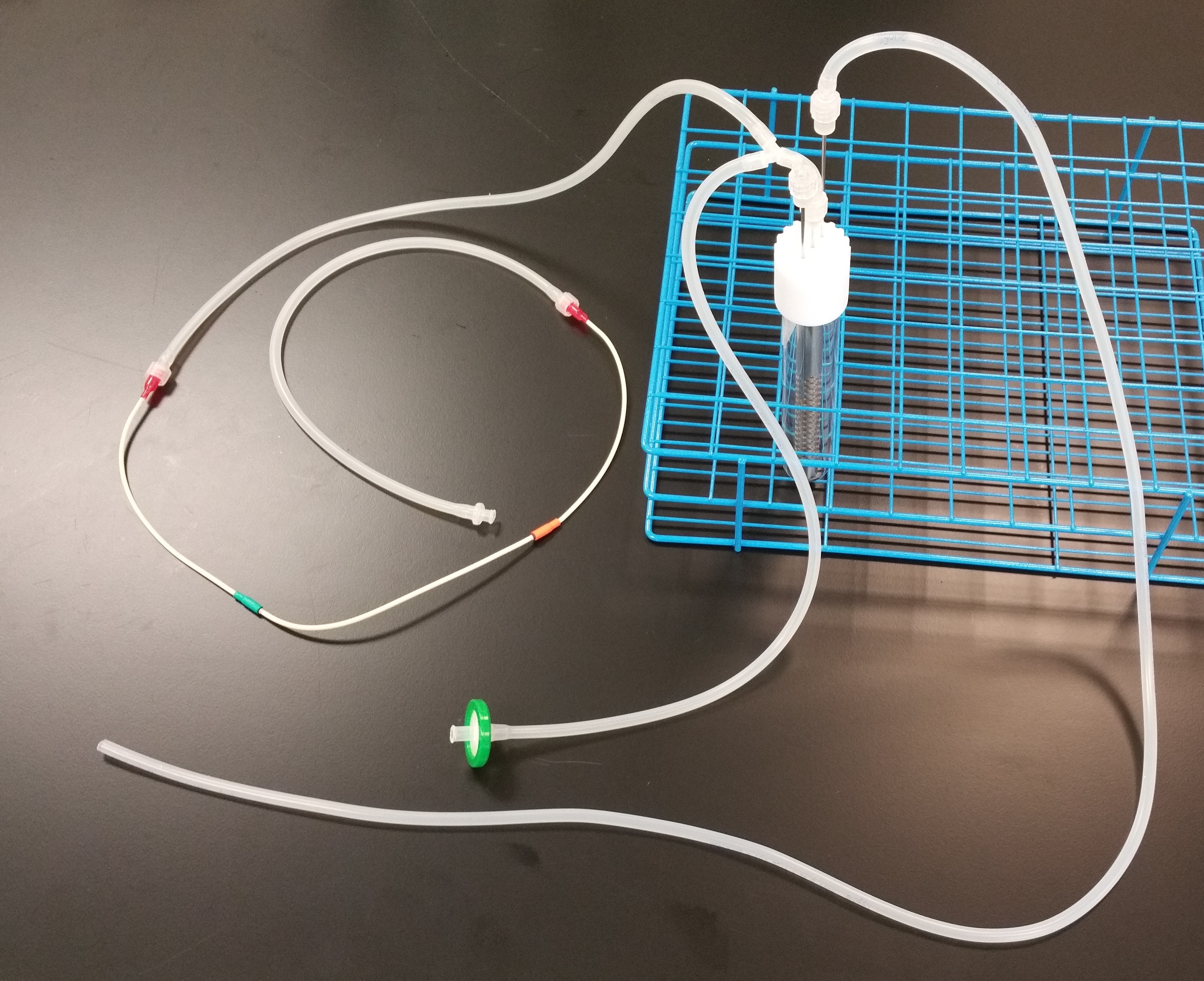

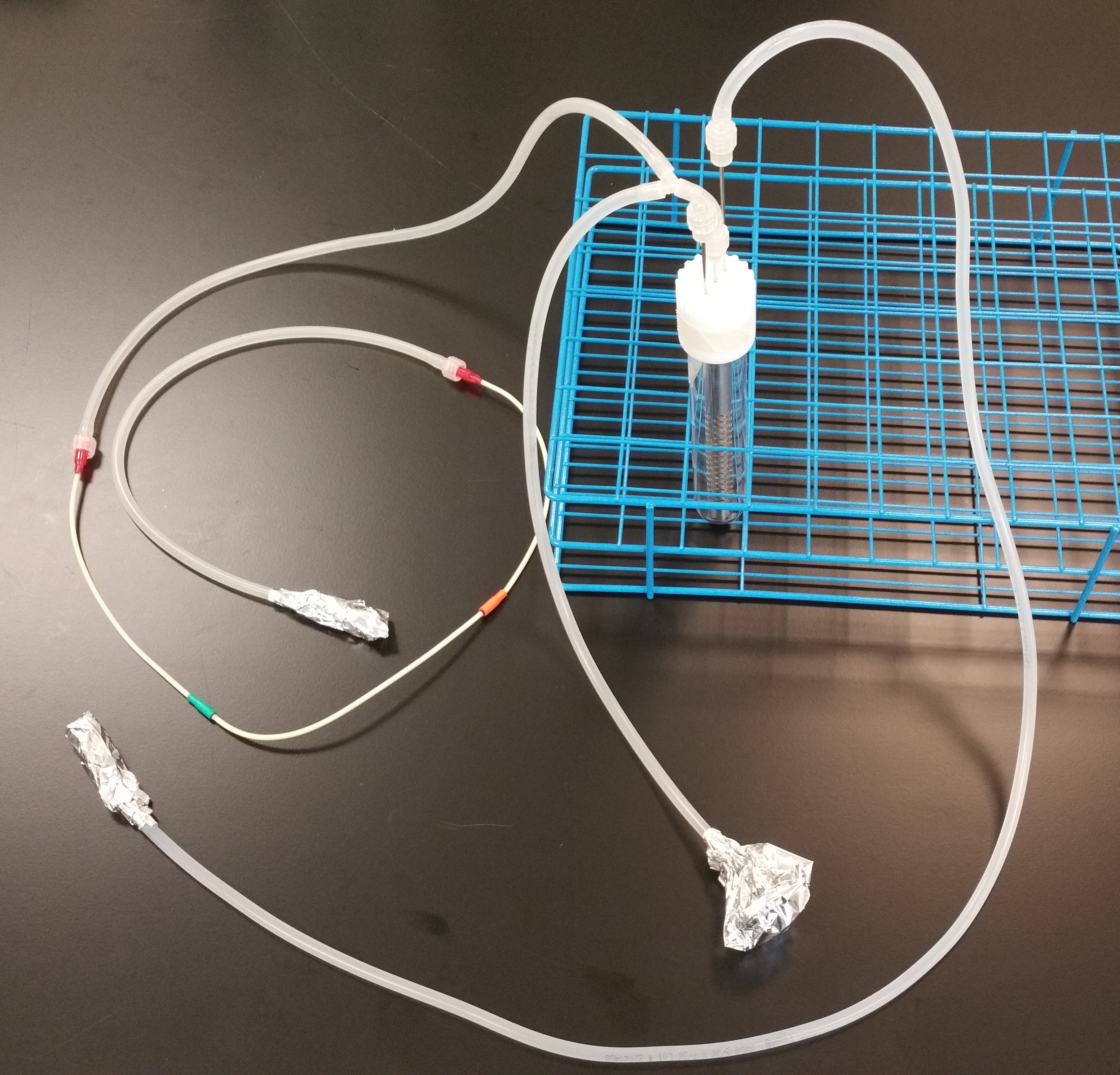


**4. Media Prep and Assembly of MAD Components**

**1) Media Prep**

Depending on the number of vessels, flow rate, and duration of MAD system running, either 10L or 2L **media carboys** can be used. 2L media is enough to run one vessel for 2-3 days. If a 10L **media carboy** is used for multiple **vessels**, prepare necessary short Y-shaped *branching tubings* to split media to 2 or more **vessels**. Normally, the tubing connected to the **media carboy** has a **male luer**, so connect a **female luer** to the end of the tubing for media-in, and **male luers** to the end of branched tubings. For the preparation of media carboy for filtration of media, please refer to *chemostat manual*, page 17-18. Before media preparation, autoclave **media carboys** (and extra Y-shaped tubings, if necessary) for 12 mins on dry cycle. Follow *chemostat manual* (page 20-26) to filter-sterilize the media.


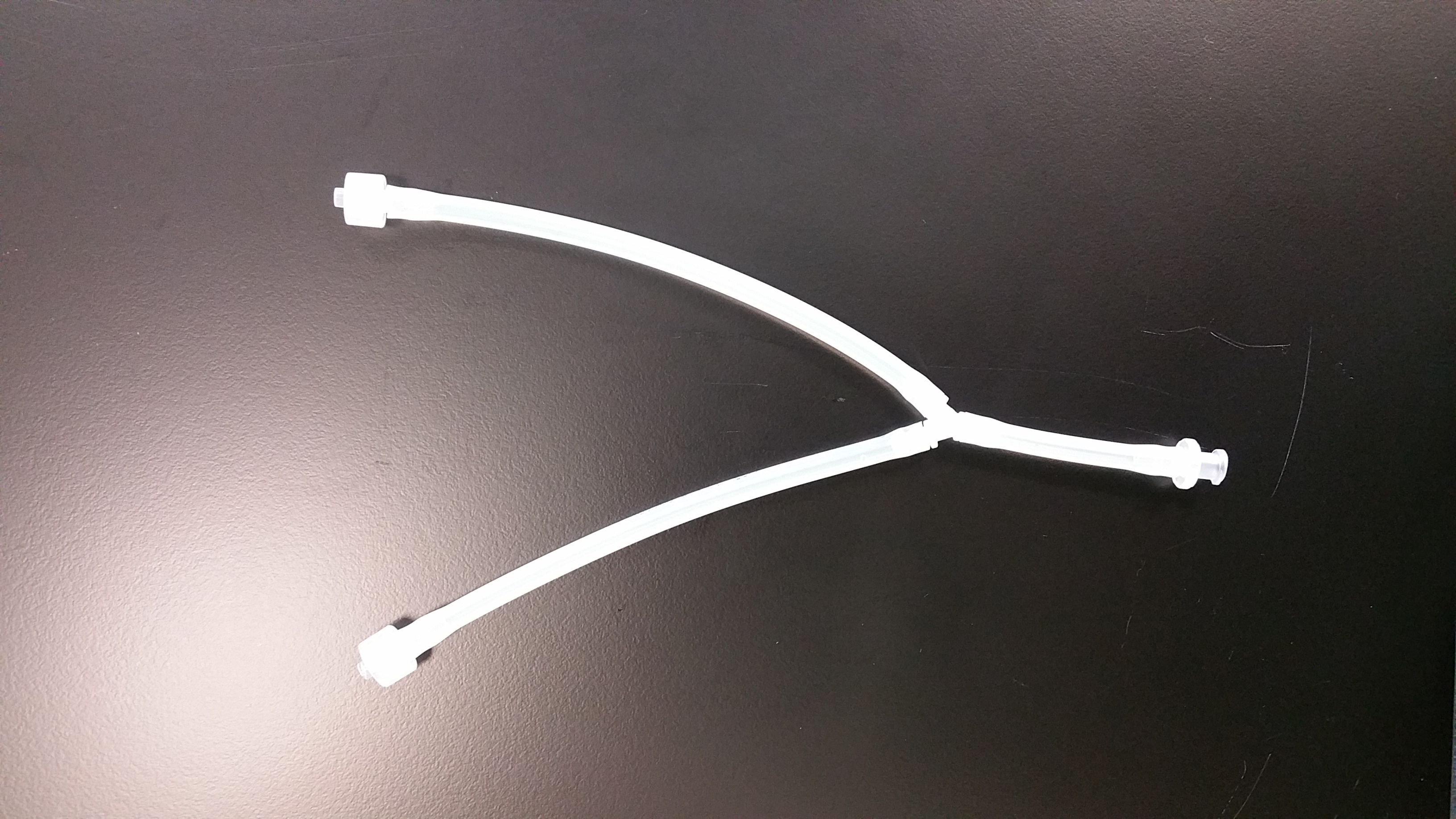


**2) Assembly of MAD Components**

- 1. Insert autoclaved vessels in the stacked **ring magnets** on the second shelf of **metro rack**.
  2. Place 1L or 2L flasks for each vessel to collect effluent on the bottom shelf of **metro rack**.
  3. First, find *effluent tubing* and guide it to the flask on the bottom shelf.
  4. Second, find and connect **air filter** to the end of **medium tubing** connected to **air-pump**.
  5. Third, find *connector tubing* with **marprene tubing**, and guide it to **peristaltic pump**. Set **marprene tubing** in snap-fit cassette with two color coded stoppers properly anchored in the cassette.
  6. Next, use 70% ethanol to sterilize your gloves and aluminum foils of a *connector tubing* and tubing from **media carboy**, and unfold aluminum foil origamis half way. Then, open both origamis and quickly connect them. If one media carboy is used for multiple vessels, connect a Y-shaped **branching tubing** between a *connector tubing* and the tubing from the **media carboy**.

**5. Running MAD System and Daily Monitoring**

**1) Peristaltic pump control**

For the **marprene tubing** with orange/green color code (bore size 0.38mm), follow the equation y=0.432x (y = flow rate [mL/hr], x = pump setting [rpm]) to set the peristaltic pump at the desired flow rate. For example, 50 RPM will pump out 25.6mL/hr and 518.4mL/day. The actual flow rate can vary from tube to tube and depending on how worn the tubing is. Fine-tuning of each vessel can be done with the “click-stop” pressure adjustment on each pump cassette.

**2) Filling in vessels with media.**

First, turn on air pumps, and remove clamps from the media carboy tubing. Second, turn on peristaltic pump and set to maximum speed (90 RPM) to fill the media in the vessel. Remove aluminum foil at the end of effluent tubing, punch a hole in the foil of the effluent collection flask, and put the tubing all the way into the flask. It will take approximately 1 hour to fill the ministat up to 40 mL. Keep running the peristaltic pump until you see effluent in the flask, and then turn off the peristaltic pump. You may keep running the ministats with air pumps overnight to make sure that there is no contamination, or you can immediately start inoculation of labeled cells.

**3) Inoculation**

Make sure that the peristaltic pump is stopped, and turn off air pump. Carefully unscrew **male lure plug** from adding port. Use P1000 pipet to inoculate labeled yeast cells.

**4) Daily Monitoring**

If tubing is not connected securely, there will be flooding of media on the system. For the first couple of hours of the system running, make sure that you do not see any leaking of media, especially from the connection points. If **air pump** pressure is set too low, there will not be enough pressure to push out excess amount of media in the **vessel**, which will result in flooding. The effluent flasks need to be large enough to house effluents overnight. It is good to cover effluent collection flask with an aluminum foil, and punch a small hole for *effluent tubing* to reduce contamination in the flask.

**6. Disassembly of MAD Components and Cleanup**

1. **Disassembly of MAD Components**
2. Turn off the peristaltic pump and air pumps. Clamp the tubing connected to **media carboy**.
3. Disconnect tubings from needles. Prepare paper towels to wipe the media, especially from tubing.
4. Disconnect all other tubing connections.

- Separate male luer(s) from female luer(s), but do not unplug luers from tubings.
- Take out marprene tubing(s) from snap-fit cassette of peristaltic pump, and separate **blunt-end needles** and **male luers**. **Blunt-end needles** can be reused after washing.
- Unplug **air filters**. These can also be reused as long as they remain dry.

1. Pull out needles from **Teflon caps**, and put them aside apart from any tubing.
2. Remove **silicon disc gaskets** from Teflon caps (using a fine forceps, if necessary). **Do not reuse these gaskets**.
3. **Cleanup**
4. All the components should be cleaned up without any detergent or bleach. **Glass vessels** used in MAD protocol should be cleaned with milliQ water and a brush as soon as possible. Otherwise, magnetic beads will stick to the **glass vessels**.
5. **Air-bubble trapper** can accumulate caramelized medium if it is not properly washed before autoclaving.
6. **Small tubings** for media, air, and effluent should be cleaned with three steps (milliQ water, 70% EtOH, and milliQ water) with squeeze bottles or pressured water source.
7. **Marprene tubings** can be reused up to 10,000 hours of running in peristaltic pump after proper cleanup. Since the bore of **marprene tubing** is small, the **peristaltic pump** can be used for cleaning (5 minutes for each three step).
8. A drinking water pump with container (shown below) can help accelerate cleanup.


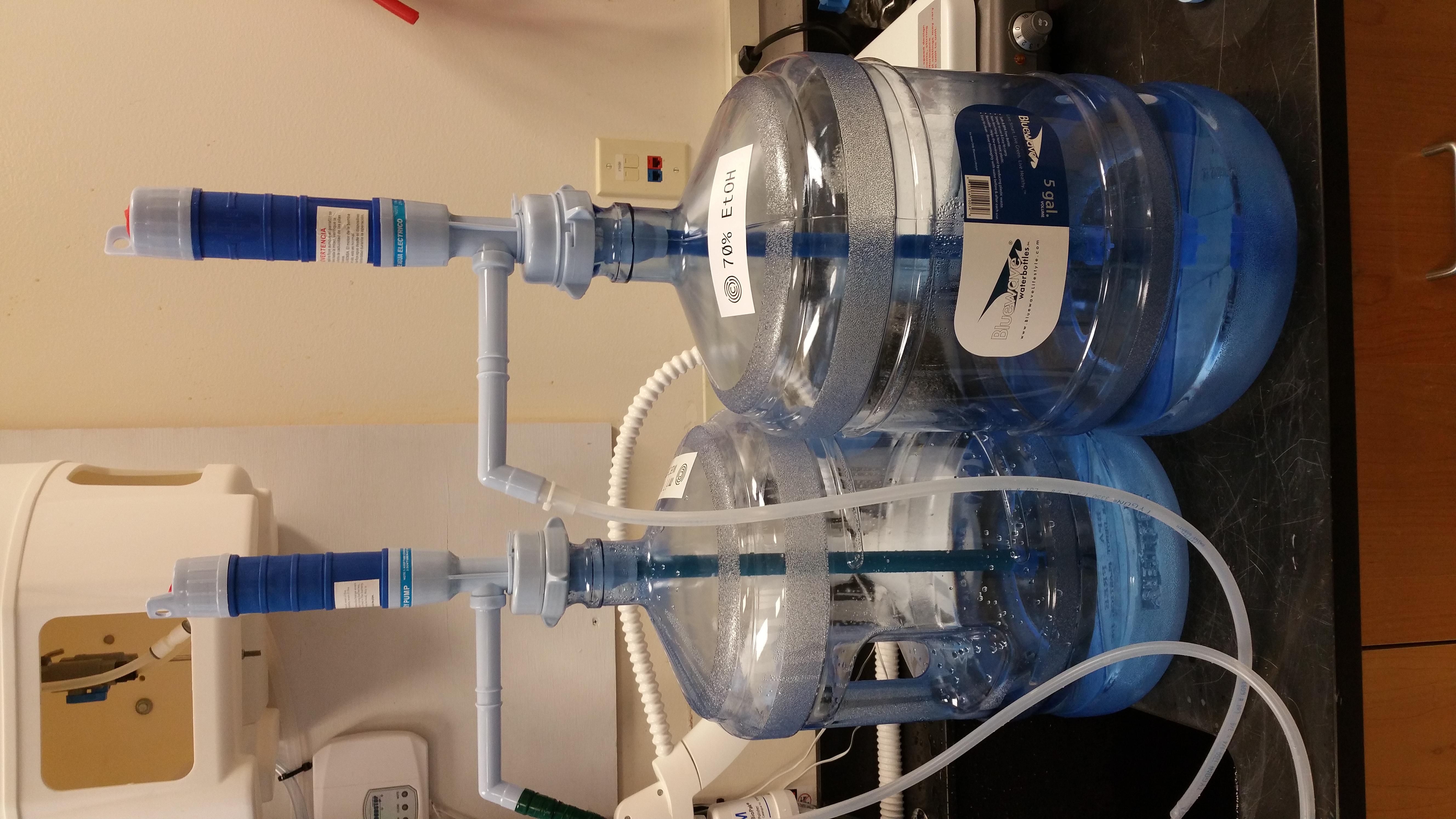

Supplement: Supplementary file 1. [file elife-39911-supp1.docx]
